# Alpha‐calcitonin gene‐related peptide prevents pressure‐overload induced heart failure: role of apoptosis and oxidative stress

**DOI:** 10.14814/phy2.14269

**Published:** 2019-11-13

**Authors:** Ambrish Kumar, Scott Supowit, Jay D. Potts, Donald J. DiPette

**Affiliations:** ^1^ Department of Cell Biology and Anatomy, School of Medicine University of South Carolina Columbia South Carolina; ^2^ Department of Internal Medicine School of Medicine University of South Carolina Columbia South Carolina

**Keywords:** Calcitonin gene‐related peptide (CGRP), cardiovascular diseases, congestive heart failure, left ventricular hypertrophy, neuropeptide, transverse aortic constriction

## Abstract

Alpha‐calcitonin gene‐related peptide (*α*‐CGRP) is a 37‐amino acid neuropeptide that plays an important protective role in modulating cardiovascular diseases. Deletion of the *α*‐CGRP gene increases the vulnerability of the heart to pressure‐induced heart failure and the administration of a modified *α*‐CGRP agonist decreases this vulnerability. Systemic administration of *α*‐CGRP decreases blood pressure in normotensive and hypertensive animals and humans. Here we examined the protective effect of long‐term administration of native *α*‐CGRP against pressure‐overload heart failure and the likely mechanism(s) of its action. Transverse aortic constriction (TAC) was performed to induce pressure‐overload heart failure in mice. We found that TAC significantly decreased left ventricular (LV) fractional shortening, ejection fraction, and *α*‐CGRP content, and increased hypertrophy, dilation, and fibrosis compared to sham mice. Administration of *α*‐CGRP‐filled mini‐osmotic pumps (4 mg/kg bwt/day) in TAC mice preserved cardiac function and LV *α*‐CGRP levels, and reduced LV hypertrophy, dilation, and fibrosis to levels comparable to sham mice. Additionally, TAC pressure‐overload significantly increased LV apoptosis and oxidative stress compared to the sham mice but these increases were prevented by *α*‐CGRP administration. *α*‐CGRP administration in TAC animals decreased LV AMPK phosphorylation levels and the expression of sirt1, both of which are regulatory markers of oxidative stress and energy metabolism. These results demonstrate that native *α*‐CGRP is protective against pressure‐overload induced heart failure. The mechanism of this cardio‐protection is likely through the prevention of apoptosis and oxidative stress, possibly mediated by sirt1 and AMPK. Thus, *α*‐CGRP is a potential therapeutic agent in preventing the progression to heart failure, and the cardio‐protective action of *α*‐CGRP is likely the result of a direct cellular effect; however, a partial vasodilatory blood pressure‐dependent mechanism of *α*‐CGRP cannot be excluded.

## Graphical abstract

Addition of native α‐CGRP prevented cardiac dysfunction due to TAC pressure‐overload. Cardiac function, hypertrophy, and reduction in oxidative stress were all brought back to control levels with α‐CGRP treatment. This presents new data on how this potent vasodilator is working to alleviate pressure‐induced heart failure.

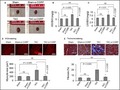

## Introduction

Alpha‐calcitonin gene‐related peptide (*α*‐CGRP) is a 37‐amino acid neuropeptide that is generated from the alternative splicing of the primary transcript of the calcitonin/*α*‐CGRP gene, CALC I (Breimer et al. 1988; Rosenfeld et al. 1983). The synthesis of *α*‐CGRP is limited to specific regions of the central and peripheral nervous system, particularly in the sensory neurons of the dorsal root ganglia. Another form of the CGRP, termed *β*‐CGRP, is encoded from a distinct gene‐CALC II, and is mainly expressed in the gut and pituitary gland (Russell et al. 2014). *α*‐ and *β*‐CGRP exerts distinct hemodynamic and gastric effects, respectively (Dubois‐Rande et al. 1992; Shekhar et al. 1991). Compared to *β*‐CGRP, *α*‐CGRP has a markedly greater activity in the regulation of cardiovascular function (Brain et al. 1985).

The cardiovascular role for *α*‐CGRP has been extensively studied in our laboratory, and others, in normal cardiovascular function and in a variety of cardiovascular diseases, including experimental hypertension, myocardial infarction, ischemic‐reperfusion cardiac injury, and heart failure (Chai et al. 2006; Gangula et al. 1997; Huang et al. 2008; Katki et al. 2001; Li et al. 2013; Li et al. 2013; Supowit et al. 2005). To date, *α*‐CGRP is the most potent vasodilator known, including the coronary circulation, and also has positive chronotropic and inotropic effects (Brain et al. 1985; Supowit et al. 1995). Systemic administration of *α*‐CGRP decreases blood pressure in normotensive and hypertensive animals and humans (DiPette et al. 1989; Supowit et al. 1993; Dubois‐Rande et al. 1992). Our laboratory has previously shown that pressure‐overload heart failure, induced by transverse aortic constriction (TAC), significantly exacerbates cardiac hypertrophy and subsequent cardiac dilation and dysfunction, cardiac fibrosis, and mortality in *α*‐CGRP knock‐out (KO) mice compared to their counterpart TAC wild‐type mice (Li et al. 2013). The hearts of TAC *α*‐CGRP KO mice exhibit a dramatic increase in apoptosis, fibrosis, inflammation, and higher collagen content, in comparison to TAC wild‐type mice, indicating that *α*‐CGRP is critical for cardio‐protection from pressure‐overload induced congestive heart failure (CHF). Our laboratory has also demonstrated that *α*‐CGRP acts as a compensatory depressor to attenuate the rise in blood pressure in three different models of experimental hypertension: (1) deoxycorticosterone (DOC)‐salt (Supowit et al. 1997), (2) subtotal nephrectomy‐salt (Supowit et al. 1998), and (3) L‐NAME induced hypertension during pregnancy (Gangula et al. 1997). A similar compensatory depressor role of *α*‐CGRP has also been shown in the two‐kidney one‐clip model of hypertension (Supowit et al. 1998) and in chronic hypoxic pulmonary hypertension (Bivalacqua et al. 2002).

Various in vivo and in vitro studies confirm that *α*‐CGRP benefits the heart by decreasing angiotensin II activity, increasing cardiac blood flow, and protecting cardiac cells from ischemia and metabolic stress (Russell et al. 2014). A recent study using rodent models of hypertension and heart failure demonstrated that the systemic subcutaneous administration of an *α*‐CGRP analogue (an acylated form of *α*‐CGRP with extended half‐life, *t*_1/2_ = ~7h) reversed the renal, vascular, and cardiac damage caused by angiotensin II‐induced hypertension or by abdominal aortic constriction (AAC)‐induced heart failure (Aubdool et al. 2017). Acylated‐*α*‐CGRP lowered the blood pressure, and reduced cardiac fibrosis, oxidative stress, and cardiac hypertrophy in these mice with hypertension and heart failure. These results indicated that *α*‐CGRP, either in its native or modified form, may benefit the patient suffering from cardiac failure. However, the protective action of the native peptide form of *α*‐CGRP in cardiovascular diseases has not been studied and the potential cellular mechanisms involved have not been determined.

The present study was undertaken to determine whether the long term administration of native *α*‐CGRP is cardio‐protective against pressure‐overload induced congestive heart failure (CHF). Here, we report that such administration of the native *α*‐CGRP peptide markedly preserved cardiac function which was accompanied by reduced apoptosis and oxidative stress, and thus protecting heart from pressure‐induced CHF.

## Materials and Methods

Eight‐week‐old male C57/BL6 mice were purchased from Charles River Laboratories, Wilmington, MA, and were housed in the institutional animal facility maintained at 25°C with a 12 h light/dark cycle. Mice received a standard diet and tap water ad libitum. Mice were allowed to acclimate for one week before the start of experiments. The animal protocols were in accordance with the guidelines of the National Institutes of Health (NIH), USA, and were approved by the University of South Carolina‐Institutional Animal Care and Use Committee.

### Pressure‐overload model

Mice were subjected to transverse aortic constriction (TAC) surgery using a 27‐gauge needle to develop pressure‐overload induced heart failure (Li et al. 2013). Briefly, mice were anesthetized with 3% isoflurane and maintained with 1% isoflurane. The chest was opened by making an incision at the suprasternal notch, and aortic constriction was performed by tying a 7‐0 silk suture ligature around a 27‐gauge needle and then removing the needle yielding 70–80% constriction. The chest was then closed and the mice were allowed to recover. Sham‐operated mice underwent the same procedure, but without aortic constriction. Two days post‐surgery, mice were divided into four groups: sham (*n* = 7), sham‐*α*‐CGRP (*n* = 7), TAC‐only (*n* = 6), and TAC‐*α*‐CGRP (*n* = 7). In the TAC‐*α*‐CGRP and sham‐*α*‐CGRP groups of mice, *α*‐CGRP‐filled osmotic minipumps (Model 1007D; Alzet Durect Corporation, CA) were implanted subcutaneously to deliver 4 mg/kg b.w/day per mouse of *α*‐CGRP (Bachem Americas Inc, CA) (*α‐*CGRP release rate = ~0.3 pmole per second). On every seventh day, new *α*‐CGRP‐filled osmotic pumps were implanted for total 28 days of *α*‐CGRP delivery. During the course of experiment, none of the sham mice died. Three of the TAC mice died within 24 h following the TAC procedure due to operative complications. After 2 days post‐TAC procedure, all of the remaining animals, for example, TAC‐only mice, and TAC and sham mice receiving *α*‐CGRP remained alive until the end of the experiment.

At the end of the experiment (day 28 of *α*‐CGRP delivery), mice were weighed and euthanized. Hearts and lungs were removed, photographed, and the wet heart and lung weight were measured. Apical portion of the heart left ventricle (LV) was snap frozen in liquid nitrogen and stored at −80°C for biochemical analyses, and basal portion was fixed in 4% paraformaldehyde/PBS (pH 7.4) for histology.

### Transthoracic echocardiography

Echocardiography was performed using Vevo 3100 High‐Resolution Imaging System (VisualSonics Inc, Toronto, Canada). Mice were sedated with 3% isoflurane and heart rate was maintained at 450–460 beats per minute by adjusting isoflurane concentration 1–1.5%. Short axis B‐ and M‐mode 2D echocardiograms were recorded through the anterior and posterior LV walls at the level of the papillary muscle. Left ventricular internal diameter at end‐systole (LVIDs) and end‐diastole (LVIDd), left ventricular posterior wall thickness, end‐systole (LVPWs) and end‐diastole (LVPWd), and fractional shortening (FS) and ejection fraction (EF) were measured by the VisualSonics Measurement Software.

### Histopathology

Paraformaldehyde‐fixed, paraffin‐embedded LV sections (5 *μ*m) were stained with Masson’s trichrome‐collagen staining (Polyscientific, Bay Shore, NY) to measure for cardiac fibrosis, and Texas Red‐X conjugated wheat germ agglutinin (WGA; Invitrogen Corp, Carlsbad, CA) staining for cardiomyocyte cross‐sectional area. For immunofluorescence, paraformaldehyde‐fixed paraffin‐embedded LV sections (5 *μ*m) were deparaffinized in xylene, rehydrated with graded ethanol (100%, 95%, and 70%) (Kumar et al. 2011). Antigen unmasking was carried out by boiling slides in 10 mmol/L sodium citrate buffer (pH 6.0) at 95 ˚C for 30 min. After permeabilization with 0.2% Triton X‐100/PBS for 10 min and blocking with 10% BSA IgG‐free/PBS (Jackson ImmunoResearch Laboratories, West Grove, PA) for overnight at 4°C, sections were incubated with primary antibodies diluted in 5% IgG‐free BSA/PBS (1:200 dilution) for overnight at 4°C. Primary antibodies were detected with secondary antibodies conjugated with Alexafluor‐488 or Alexafluor‐546 (Invitrogen). DAPI (4′, 6‐diamidino‐2‐phenylindole; Sigma‐Aldrich, St. Louis, MO) was used to stain nuclei. Sections were mounted with antifade Vectashield mounting media (Vector Laboratories, Burlingame, CA), and signals were visualized under Nikon‐E600 fluorescence microscope. Primary antibodies were: cleaved caspase‐3 (Cell Signaling Technology, Danvers, MA), 8‐OHdG, sirt1 (Santa Cruz Biotechnology, Santa Cruz, CA), and anti‐HNE (4‐hydroxy‐2‐nonenal; Abcam Inc, Cambridge, MA). NIH‐ImageJ software (USA) was used to quantitate collagen content, cardiomyocyte area, and fluorescence intensity in the left ventricles.

### TUNEL staining

DeadEnd fluorometric TUNEL kit (Promega, Madison, WI) was used to detect apoptotic DNA fragmentation in the LV sections. Briefly, LV sections (5 *µ*m thick) were deparaffinized in xylene, rehydrated with ethanol, and washed with 0.85% NaCl. Tissue sections were fixed with 4% para‐formaldehyde/PBS solution followed by permeabilization with proteinase K solution (20 *µ*g/mL) for 10 min at room temperature. The nicked DNA was labeled with fluorescence‐labeled dUTP nucleotide and recombinant terminal deoxynucleotidyl transferase enzyme mix for 60 min at 37°C. After washing with 2× standard saline citrate, sections were mounted with Vectashield mounting media (Vector Laboratories) and examined under Nikon‐E600 fluorescence microscope. NIH‐ImageJ software was used to count apoptotic cells in 20 random fields.

### Western blotting

Total protein from the LVs was extracted by 1× RIPA cell lysis buffer (Cell Signaling Technology), and stored at −80°C until use. Protein concentrations were measured by bicinchoninic acid protein assay kit (Pierce/ThermoScientific, Waltham, MA). Equal amount of extracted proteins were diluted with 5× Laemmli sample loading buffer, boiled for 5 min, separated by SDS‐polyacrylamide gel electrophoresis, and analyzed by western blotting (Kumar et al. 2014). Briefly, after electrophoresis, proteins were transferred on polyvinylidene difluoride (PVDF) membrane at 100 volt for 3 h in cold room. Membrane was blocked with 10% non‐fat dry milk/TBST (20 mmol/L Tris‐Cl, pH 7.4; 150 mmol/L NaCl with 0.1% Tween‐20) for 4 h at room temperature followed by incubation in primary antibodies diluted in 5% non‐fat dry milk/TBST for overnight at 4°C. After washing with TBST, membrane was incubated with corresponding horseradish peroxidase‐conjugated secondary antibodies (Bio‐Rad Laboratories, Hercules, CA) diluted in 5% non‐fat dry milk/TBST for 2 h at room temperature. Signals were detected by Clarity Western Detection Kit (Bio‐Rad Laboratories). Primary antibodies used were Superoxide dismutase ‐2 (SOD‐2), *β*‐actin, phospho‐AMPK^Thr172^, and total‐AMPK (all from Cell Signaling Technology). The expression of these proteins was quantitated by NIH‐ImageJ software.

### Enzymatic activity assays

Total glutathione (GSH) content in the LVs was measured by luminescence based GSH‐Glo Glutathione assay kit (Promega), and generated luminescence, corresponding to glutathione level, was detected by Turner 20/20 luminometer (Promega). Lipid peroxidation assay kit (Sigma) was used to measure malondialdehyde (MDA) level, an indicator of lipid‐peroxidation, in the LVs.

### Measurement of LV *α‐*CGRP content

Enzyme‐linked immunosorbent assay (ELISA) was performed to measure LV *α‐*CGRP level using an *α‐*CGRP EIAH kit (Peninsula Laboratories, San Carlos, CA). Briefly, LV tissues were boiled and homogenized in 5% acetic acid solution. After centrifugation at 12,000*g* for 10 min, the clear supernatant was collected and peptides were extracted through C18 Sep‐column. The extracted peptides were lyophilized, suspended in EIA buffer, and 50 *μ*g of extracted peptide was used for ELISA following manufacturer’s protocol II instructions. Absorbance was measured at 450 nm in Spectramax Plus 384 microplate reader (Molecular Devices, CA).

### Statistical analysis

Data were expressed as mean ± SD. Comparisons were made among the groups using one‐way ANOVA followed by Tukey‐Kramer ad hoc test (GraphPad software, La Jolla, CA). *P* value <0.05 was considered significant.

## Results

### *α‐*CGRP content in the left ventricle and serum

**Figure 1:**
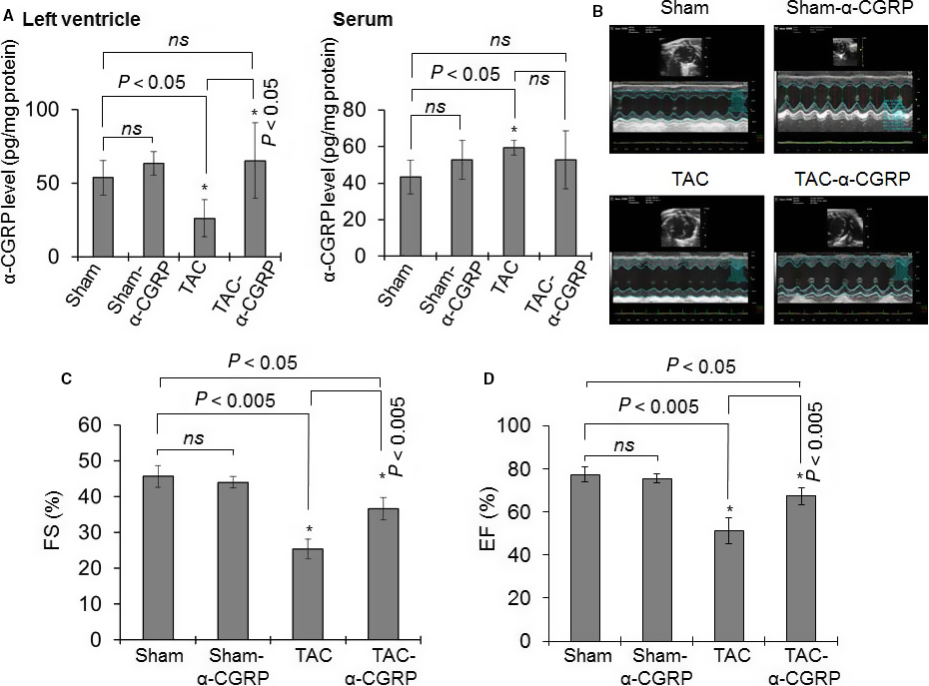
(A) Bar diagrams showing *α*‐CGRP content in the left ventricle (LV) and serum collected from sham, sham‐*α*‐CGRP, TAC, and TAC‐*α*‐CGRP mice after 28 days *α*‐CGRP delivery. Values were expressed as the mean ± SD. (B) Representative echocardiograms showing short axis B‐ and M‐mode 2D echocardiography performed after *α*‐CGRP delivery (day 28). (C and D) Percentage fractional shortening (FS) and ejection fraction (EF) were plotted as the mean ± SD. *P < 0.05 was considered significant. *ns* = non‐significant.

ELISA was performed to measure *α‐*CGRP levels in the LVs and serum after 28 days of sustained peptide delivery. The measured LV *α‐*CGRP content in sham, sham‐*α*‐CGRP, TAC, and TAC‐*α*‐CGRP mice were 53.7 ± 11.9, 63.5 ± 8.3, 26.2 ± 12.5, and 65.35 ± 25.6, (in pg/mg protein ± SD), respectively (Fig. 1A). The LV *α‐*CGRP level in the TAC mice was significantly lower compared to with the sham‐operated mice (*P* < 0.05, TAC vs. sham). In addition, *α‐*CGRP levels were significantly higher in the TAC‐*α*‐CGRP mice than in TAC mice (*P* < 0.05, TAC‐*α*‐CGRP vs. TAC). Although the LV *α‐*CGRP level in TAC‐*α*‐CGRP mice and sham‐α‐CGRP mice was higher compared to the sham mice, interestingly this increase was not statistically significant (Fig. 1A).

ELISA assay conducted in serum collected from the sham, sham‐*α*‐CGRP, TAC, and TAC‐*α‐*CGRP group of mice demonstrated that *α‐*CGRP content in TAC mice serum was significantly higher than that of sham group (*P* < 0.05, TAC vs. sham). No significant difference in *α‐*CGRP levels between sham, sham‐*α‐*CGRP, and TAC‐*α‐*CGRP groups was observed (Fig. 1A). It is possible that the circulating level of *α*‐CGRP administered by the osmotic‐minipump in the TAC‐*α*‐CGRP and sham‐*α*‐CGRP groups of mice was exhausted by the end of the study; which account for the minimal change in peptide level in these groups of mice compared to sham‐operated mice.

### Exogenous *α*‐CGRP administration improves cardiac function in TAC mice

Our laboratory has previously shown that TAC significantly exacerbates cardiac hypertrophy and subsequent cardiac dilation and dysfunction in *α*‐CGRP knock‐out (KO) mice (Li et al. 2013). To determine if long‐term administration of *α*‐CGRP preserved cardiac function, B‐ and M‐mode 2D electrocardiography was performed on day 28 following *α*‐CGRP delivery in all four groups (Fig. 1B–D). LV systolic function, as assessed by % fraction shortening (FS; Fig. 1C) and ejection fraction (EF; Fig. 1D), was significantly decreased in the TAC mice compared to the sham mice. Percent FS in sham and TAC mice were (in ± SD) 45.7 ± 3 and 25.4 ± 2.8, respectively, (*P* < 0.005 sham vs. TAC). Compared to TAC‐only mice, the administration of *α*‐CGRP significantly preserved FS in the TAC‐*α*‐CGRP mice (%FS ± SD: TAC‐*α*‐CGRP = 36.6 ± 3.0, *P* < 0.005 TAC vs. TAC‐*α*‐CGRP) and changes in EF were similar to changes in FS where the reduction in %EF was preserved in the TAC‐*α*‐CGRP mice compared to the TAC mice (*P* < 0.005, TAC‐*α*‐CGRP vs. TAC). Although the administration of *α*‐CGRP markedly preserved both FS and EF in the TAC‐*α*‐CGRP mice compared to the TAC mice, both parameters were lower than that observed in the sham‐operated mice. Our results also demonstrated that FS and EF values were not significantly different in sham and sham‐*α*‐CGRP groups. These results indicate that *α*‐CGRP administration preserved cardiac function in pressure‐overload induced heart failure.

### *α*‐CGRP administration attenuates cardiac hypertrophy and fibrosis in TAC mice

**Figure 2:**
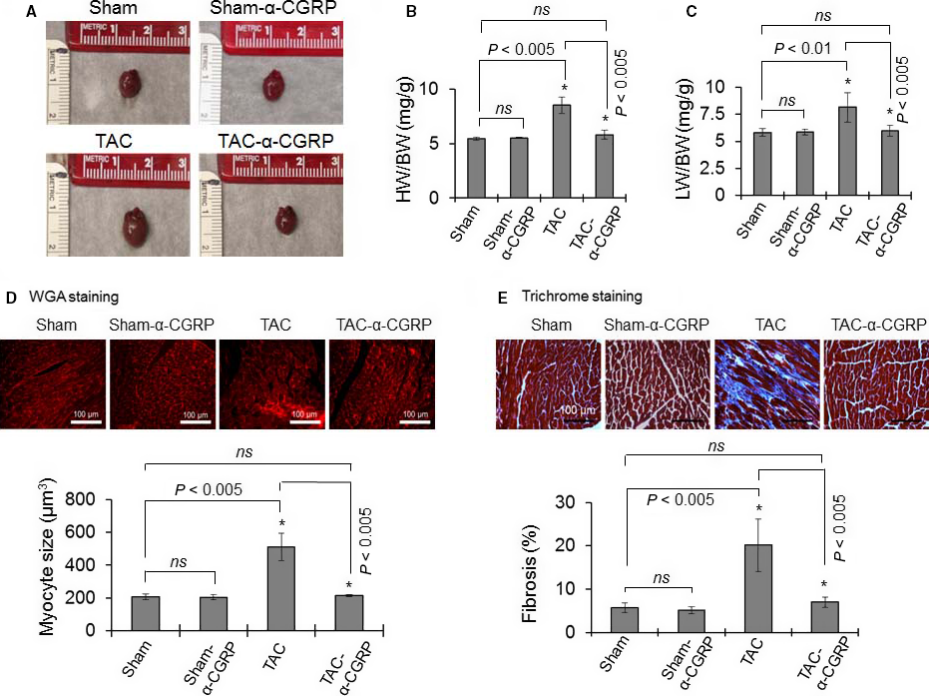
(A) Representative photographs showing the size of the hearts of sham, sham‐*α*‐CGRP, TAC, and TAC‐*α*‐CGRP mice after 28 days *α*‐CGRP delivery. (B and C) Bar diagrams represent the ratio of wet heart weight/body weight (HW/BW), and wet lung weight/body weight (LW/BW). (D) The paraformaldehyde‐fixed, paraffin‐embedded LV sections (5 µm) were stained with WGA stain (upper panel). Cell size was measured by NIH‐ImageJ software and plotted (lower panel). Scale bar = 100 *μ*m. (E) Trichrome‐collagen stained LV sections (upper panel) were used to measure fibrosis. Collagen content, an indicator of fibrosis, was quantitated by NIH‐ImageJ software and plotted (lower panel). Values were expressed as the mean ± SD. *P < 0.05 was considered significant. *ns* = non‐significant.

To further determine the effect of *α*‐CGRP on TAC induced cardiac hypertrophy and fibrosis, isolated hearts at 28 days were photographed and the ratios of wet heart weight to body weight (HW/BW) and wet lung weight to body weight (LW/BW) were measured as indices of LV hypertrophy and dilation and pulmonary congestion (Fig. 2A–C). The sizes of the TAC‐hearts were larger than that of sham‐hearts, while the heart size among the TAC‐*α*‐CGRP mice and sham mice appeared identical (Fig. 2A). The calculated mean HW/BW was significantly greater in TAC mice compared to sham mice (*P* < 0.005, TAC vs. sham) while the increase in HW/BW after TAC was significantly attenuated by *α*‐CGRP administration (*P* < 0.005, TAC‐*α*‐CGRP vs. TAC; Fig. 2B). Similarly, the LW/BW was significantly increased in TAC mice (*P* < 0.01, TAC vs. sham) which was significantly attenuated by *α*‐CGRP in the TAC‐*α*‐CGRP mice (*P* < 0.005, TAC vs. TAC‐*α*‐CGRP; Fig. 2C). The heart size and the ratios HW/BW and LW/BW in the sham‐*α*‐CGRP group of mice were similar to that of the sham‐operated mice (Fig. 2A–C).

Wheat germ agglutinin (WGA) staining and its quantitation (Fig. 2D, upper and lower panel) showed that the size of myocytes in TAC LV was markedly increased compared to their sham counterparts (*P* < 0.005, TAC vs. sham). In comparison, TAC‐*α*‐CGRP LV myocytes were similar size in to that seen in the sham mice (*P* < 0.005, TAC‐*α*‐CGRP vs. TAC). In addition, the size of LV myocytes in the sham‐only and sham‐*α*‐CGRP groups of mice was similar. Furthermore, LV interstitial fibrosis, as determined by trichrome‐collagen staining, was significantly greater in the TAC mice compared to sham‐operated mice (*P* < 0.005, TAC vs. sham). Similarly, this increase was significantly attenuated by *α*‐CGRP administration in TAC mice and was similar to that seen in the sham mice (*P* < 0.005, TAC vs. TAC‐*α*‐CGRP, Fig. 2E, upper and lower panel). In the sham‐*α*‐CGRP mice, we observed little fibrosis in the LVs and LV collagen content was comparable to sham‐only mice (Fig. 2E).

### *α*‐CGRP administration inhibits apoptosis in the TAC left ventricle

**Figure 3:**
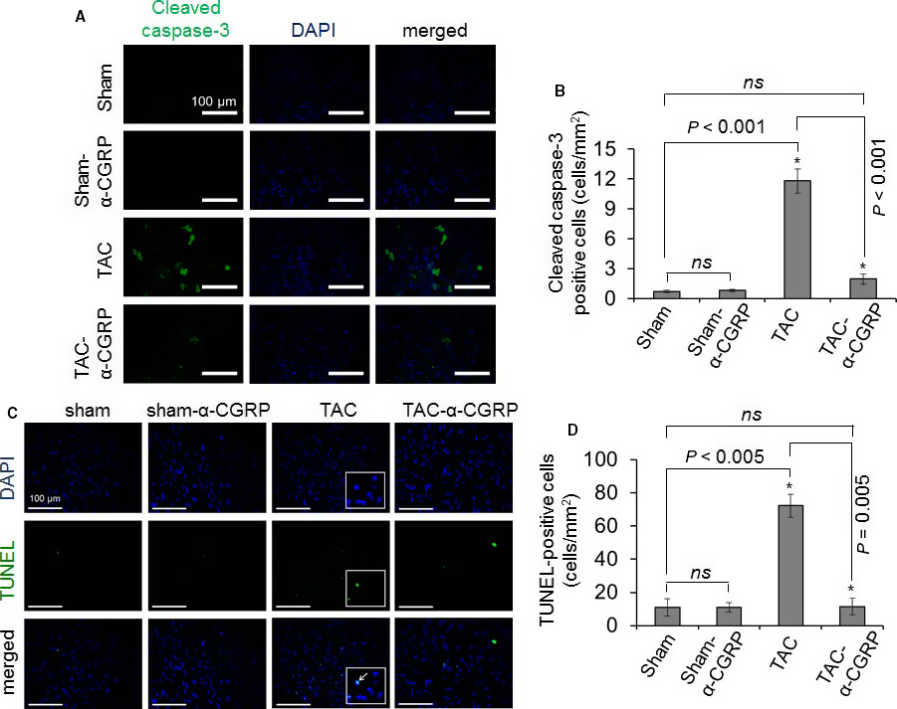
Representative fluorescence images showing cleaved caspase‐3 staining (A) and TUNEL‐staining (C) to detect apoptosis in the LV sections. DAPI was used to stain nuclei. Cleaved caspase‐3 (cytoplasmic, green in color) and TUNEL‐positive cells (nuclear, green in color) were counted and plotted as the mean ± SD (B and D). Enlarged image boxes in TAC LVs were showing the overlapping of TUNEL‐DNA fragmentation staining and DAPI‐nuclear staining in a single cell (white arrow, Fig. 3C). Scale = 100 *μ*m.*P < 0.05 was considered significant. *ns* = non‐significant.

Because apoptosis is one possible mechanism for the ultimate failure seen in the TAC animals, staining for apoptotic markers was performed. The immunofluorescent images in Figure 3A demonstrated that the number of cleaved caspase‐3 positive cells (green), an indicator of apoptotic cell death, were higher in TAC LVs compared to the sham LV (*P* < 0.001, TAC vs. sham; Fig. 3B). In comparison, the number of cleaved caspase‐3 positive cells was significantly lower in the TAC‐*α*‐CGRP LV compared to TAC‐only LV samples (*P* < 0.001, TAC‐*α*‐CGRP vs. TAC; Fig. 3A and B).

To further examine the apoptotic pathway in the hearts, apoptotic DNA fragmentation was determined by TUNEL assay. In sections stained for TUNEL positive cells, images showed that compared to the sham‐operated mice, the number of TUNEL‐positive cells (green) was significantly greater in TAC LVs (sham = 11 ± 3 cells/mm^2^, TAC = 72 ± 3 cells/mm^2^; *P* < 0.005, TAC vs. sham) (Fig. 3C and D). The staining of nuclei of TUNEL‐positive cells in TAC LV were superimposed in a single cell (shown in enlarged images in TAC LVs in Fig. 3C). Compared to the TAC LV, the number of TUNEL‐positive cells in the TAC‐*α*‐CGRP LV was significantly less (TAC‐*α*‐CGRP = 11 ± 4 cells/mm^2^; *P* < 0.005, TAC vs. TAC‐*α*‐CGRP). The number of cleaved caspase‐3 positive cells (Fig. 3B) and TUNEL‐positive cells (Fig. 3D) between sham and sham‐*α*‐CGRP LVs was not significantly different. These results indicated that *α*‐CGRP administration protects LV cardiac cells from pressure‐overload induced apoptosis.

### *α*‐CGRP protects the LVs from oxidative stress generated by TAC

**Figure 4:**
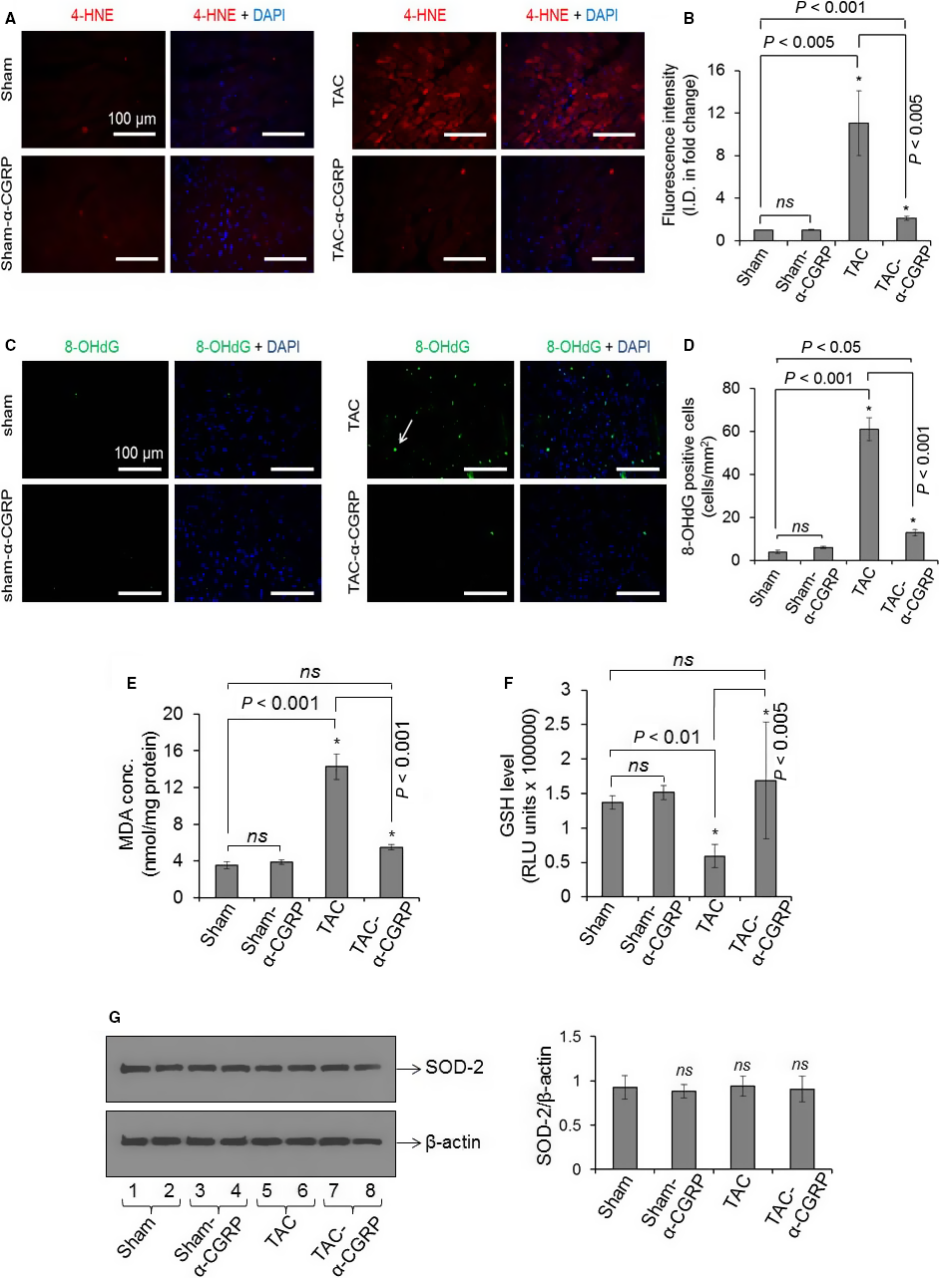
Representative fluorescence images showing 4‐HNE staining (a marker of lipid peroxidation; Fig. 4A), and 8‐OHdG staining (a marker of oxidative DNA damage; Fig. 4C) in the LV sections. DAPI was used to stain nuclei. White arrow in Figure 4C showed 8‐OHdG stained cell (green) in the TAC LVs. Scale = 100 *μ*m. (B and D) The fluorescence intensity of 4‐HNE (red), and number of 8‐OHdG stained cells (green) in the LV sections were quantitated by NIH‐ImageJ software and plotted as the mean ± SD. I.D. = Integrated density. Bar diagrams showing malondialdehyde (MDA) concentration (E) and glutathione (GSH) level (F) in the LV of sham, sham‐*α*‐CGRP, TAC, and TAC‐*α*‐CGRP mice after *α*‐CGRP delivery (28 days). Values were expressed as the mean ± SD and *P < 0.05 was considered significant. *ns* = non‐significant. (G) Western blots showing SOD‐2 and *β*‐actin protein level in total cell proteins extracted from sham, sham‐*α*‐CGRP, TAC, and TAC‐*α*‐CGRP LV tissues. The ratio SOD‐2/*β*‐actin was plotted as the mean ± SD. *ns* = non‐significant.

Several reports suggest that pressure‐overload induced apoptosis in LV myocytes is associated with increased oxidative stress (Burgoyne et al. 2012; Nojiri et al. 2006). Thus, to further examine the apoptotic role in heart failure, we used various oxidative stress markers to determine oxidative stress levels in the LVs. We determined that the LV expression of 4‐HNE (lipid‐peroxidation marker) and 8‐OHdG (oxidative DNA damage marker) was significantly increased in the TAC mice compared to their sham counterpart. And *α*‐CGRP administration significantly reduced the formation of HNE‐adduct and attenuated the increase in 8‐OHdG in the TAC‐*α*‐CGRP LVs. Although the levels of 4‐HNE and 8‐OHdG were significantly lower in the TAC‐*α*‐CGRP LV, they were increased when compare to the sham LV levels (Fig. 4A–D). The malondialdehyde (MDA) generation, a marker of oxidative stress‐induced lipid peroxidation, was significantly increased in the TAC LVs while significantly lowered by *α*‐CGRP (in nmol/mg protein ± SD, sham = 3.55 ± 0.4, sham‐*α*‐CGRP = 3.9 ± 0.3, TAC = 14.27 ± 1.4, TAC‐*α*‐CGRP = 5.50 ± 0.3; *P* < 0.001 TAC vs. TAC‐*α*‐CGRP, Fig. 4E). Pressure‐overload reduced total GSH levels in the TAC LVs (*P* < 0.01 sham vs. TAC) whereas *α*‐CGRP administration restored it to the levels similar to that seen in the sham group (*P* < 0.005 TAC vs. TAC‐*α*‐CGRP, Fig. 4F). Interestingly, there was no difference in superoxide dismutase‐2 (SOD‐2) protein levels between the LVs from any group of mice (Fig. 4G). Moreover measured oxidative stress parameters in sham LVs were comparable with sham‐*α*‐CGRP LVs (Fig. 4A–G).

### *α*‐CGRP administration attenuates the increase in the nuclear expression of sirt1 and activation of AMPK in the TAC LVs

**Figure 5:**
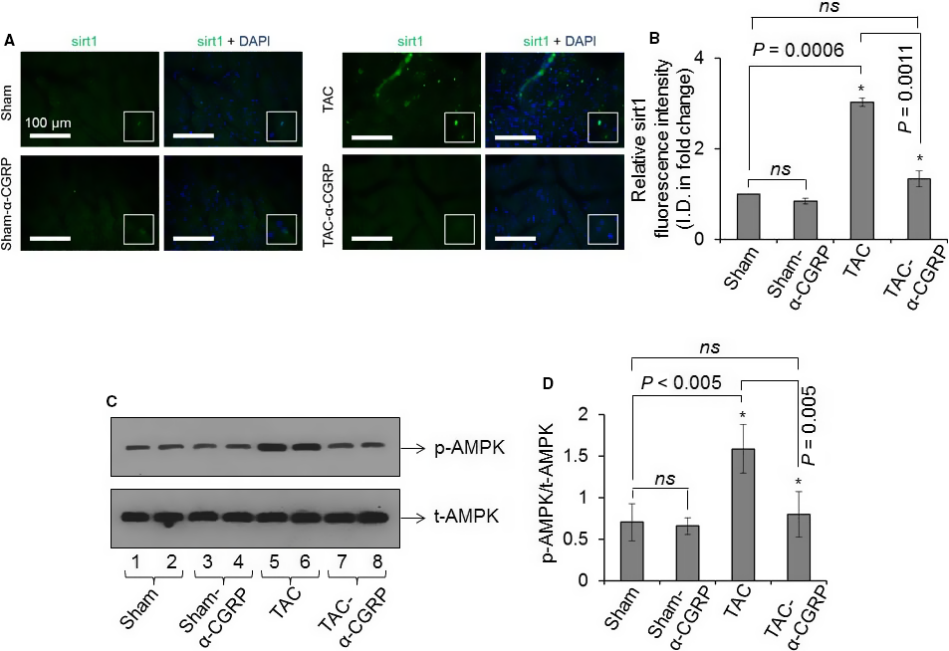
(A) Representative immunofluorescence images showing the nuclear staining of sirt1 (green) in the sham, sham‐*α*‐CGRP, TAC, and TAC‐*α*‐CGRP LV sections after *α*‐CGRP delivery (day 28). DAPI was used to stain nuclei. White boxes showed the enlarged area with sirt1 staining in the nucleus. Scale = 100 *μ*m. (B) The sirt1 fluorescence intensity was quantitated by NIH‐ImageJ software and plotted as the mean ± SD. (C and D) Western blots showing the protein level of total‐ and phospho‐AMPK in the LV of sham, sham‐*α*‐CGRP, TAC, and TAC‐*α*‐CGRP mice after *α*‐CGRP delivery (day 28). The protein band intensity was quantitated and plotted as the mean ± SD. *P < 0.05 was considered significant. *ns* = non‐significant.

Hypertrophic hearts have structurally defective mitochondria and impaired mitochondrial energy metabolism that eventually results in increased oxidative stress and cardiac cell death with resultant heart failure (Oka et al. 2011; Russell et al. 2005). Additionally, it’s been reported that pressure‐overload results in an upregulation of sirt1 which suppresses ERR transcriptional pathways regulating mitochondrial genes that, in turn, promotes mitochondrial dysfunction in the heart (Alcendor et al. 2007; Oka et al. 2011). Thus, we determined whether *α*‐CGRP could protect cardiac cells from oxidative stress through sirt1 and AMPK. Immunohistochemistry demonstrated that the fluorescence intensity of nuclear sirt1 was significantly increased in the TAC LV compared to the sham counterparts (*P* < 0.001, TAC vs. sham), and *α*‐CGRP administration attenuated this increase in nuclear sirt1 levels to sham levels (*P* < 0.005, TAC vs. TAC‐*α*‐CGRP; Fig. 5A and B). Western blot data demonstrated that the phospho‐AMPK (p‐AMPK) protein level was markedly increased in TAC LVs when compared with their sham counterparts (*P* < 0.05, TAC vs. sham), however *α‐*CGRP treated LVs from TAC‐*α*‐CGRP group of mice had levels of phospho‐AMPK which were comparable to sham mice (*P* < 0.05, TAC vs. TAC‐*α*‐CGRP). In comparison, no change in total‐AMPK (t‐AMPK) levels in the LVs between any of the three groups of mice was observed (Fig. 5C and D). These results indicate that *α*‐CGRP significantly attenuated AMPK activation in TAC LVs.

## Discussion

The aim of the present study was to determine if the continuous administration of native *α*‐CGRP is cardio‐protective in pressure‐overload induced heart failure and, if so, which cellular mechanism(s) may be responsible. The major findings of this study are that long‐term native *α*‐CGRP administration to mice with TAC: 1). prevents adverse cardiac remodeling and dysfunction; 2). significantly attenuates the left ventricular increase in oxidative stress, apoptosis, and fibrosis; 3). significantly attenuates the left ventricular increase in sirt1 and p‐AMPK. The current study together with our previous report using an *α*‐CGRP‐knock out TAC heart failure mouse model (Li et al. 2013) reconfirms that *α*‐CGRP plays an important cardio‐protective role in pressure overload‐induced CHF. These studies support and extend the positive results demonstrated with the administration of an *α*‐CGRP agonist analogue to rodents with abdominal aortic constriction (Aubdool et al. 2017).

In the present study, 28 days following TAC surgery, we found significantly lower levels of *α*‐CGRP in the LVs compared to the sham mice. Previously, our laboratory reported that on day 14 and 21 after TAC surgery, there was an increase in LV *α*‐CGRP content when compared to the sham mice. However, there was a decrease in LV *α*‐CGRP content between the 14 and 21 day time points (Li et al. 2013). Combining present and previous findings on days 14, 21, and 28 following TAC, it is evident that pressure‐overload elevates LV *α*‐CGRP content initially for the first few weeks, however as heart failure worsens, the LV *α*‐CGRP content continues to decrease to levels below that seen following sham treatment. This finding agrees with studies in humans showing that the circulating levels of *α*‐CGRP are elevated during the initial and middle stages of heart failure but then significantly decline as heart failure progresses (Dubois‐Rande et al. 1992). These data suggest that during the initial phase of heart failure, *α*‐CGRP content in the LV increases in a compensatory manner to protect against the initial insult. However, this increase in *α*‐CGRP levels cannot be sustained due to the potential further cellular necrosis which could include *α*‐CGRP producing sensory nerves. It has been reported that, during the heart failure, the cardiac cells first enlarged in an effort to compensate for the pressure overload generated by TAC (Nakamura and Sadoshima 2018). Under severe conditions, as the disease progresses, cardiac cells start to die via apoptosis and/or oxidative injury leading to myocardial cellular necrosis (Nakayama et al. 2007; van der Pol et al. 2019). During this process, *α*‐CGRP‐producing sensory nerves which are distributed in the cardiac sensory nerves including in periadvential tissue of the cardiac arteries and which course within the myocardium are potentially also destroyed by the same processes. It is possible that during the progression of cardiac failure and under prolonged stress conditions, the cardiac sensory nerves get desensitized and depleted with *α*‐CGRP as a result the release of the peptide from *α*‐CGRP positive nerves decreases over time. This notion is supported by the fact that differential release of *α*‐CGRP from the cardiac sensory nerves has been observed after repeated application of capsaicin, an agonist of TRPV1 (transient receptor potential vanilloid receptor 1) that induces the release of *α*‐CGRP from the sensory nerves (Franco‐Cereceda et al. 1989). Capsaicin induces the release of *α*‐CGRP from the sensory nerves through the activation of TRPV1, however high dose of capsaicin pretreatment causes degeneration of *α*‐CGRP‐positive sensory nerves in the myocardium that leads to marked reduction of the *α*‐CGRP release from these sensory nerves and impair cardiac function (Wharton et al. 1986; Zhang et al. 2012). Overall, lower levels of cardiac *α*‐CGRP in the TAC‐LVs on day 28 might be a result of reduced release of *α*‐CGRP due to depletion of the stored peptide in the sensory nerve or by cellular death of these nerves in the heart.

Our previous publication showed that TAC pressure overload further enhanced angiogenesis and inflammation in *α*‐CGRP KO mice (Li et al. 2013). We speculate that *α*‐CGRP delivery might inhibit inflammation and prevent angiogenesis in TAC‐heart. We have not tested these scenarios; however, we will test the possible role of these pathways and other signaling mechanism in the future.

It is well established that in the failing heart increased oxidative stress promotes apoptotic cell death (Burgoyne et al. 2012; Nojiri et al. 2006). Increased lipid peroxidation (4‐HNE and MDA level) and oxidative DNA damage (8‐OHdG level), and decreased GSH content in the TAC LVs, as seen in present study, suggest that aortic constriction‐induced pressure‐overload generates ROS and thus oxidative stress that in turn enhance apoptotic cell death in the LVs. Under normal conditions, excess ROS are detoxified by the GSH to prevent cell death (Kannan and Jain 2000). The reduced level of GSH in the TAC LV suggests that detoxifying enzymes are exhausted and on their own unable to remove the excess ROS generated under this stress conditions. Our studies showed that *α*‐CGRP delivery significantly lowers lipid peroxidation and oxidative DNA damage in the TAC‐*α*‐CGRP LVs. Moreover *α*‐CGRP treatment prevented TAC‐induced depletion of intracellular GSH level in the LV. It is possible that the toxic HNE by‐products produced following TAC have been detoxified by the preserved levels of GSH, thereby protecting cells from apoptosis induced by oxidative stress. Alternatively, since the oxidative stress following TAC was prevented by *α*‐CGRP administration, GSH involvement was not critical. It has been reported that nrf‐2 signaling pathway, a protective anti‐oxidative mechanism, maintains the cellular redox homeostasis via upregulating expression of several detoxifying enzymes, including superoxide dismutase (SOD‐2), catalase, heme oxygenase 1 (HO‐1), and glutathione peroxidase (Niture et al. 2014). However, we did not find any significant differences in the levels of nrf‐2, Hif‐1 (data not shown) or SOD‐2 between any of the groups. These observations are consistent with studies demonstrating that following TAC procedure the expression of nrf‐2 and nrf‐2 downstream genes were increased initially and subsequently decreased to nearly the basal level on day 28 (Li et al. 2009), coinciding to the time point of our study. Thus, the *α*‐CGRP cardio‐protective effects seen do not appear to be totally mediated by the nrf‐2 pathway.

Pressure‐overload is known to impair mitochondrial energy metabolism in hypertrophic hearts, and the proteins sirt1 (a NAD^+^‐dependent histone deacetylase) and AMPK act as energy sensors under mitochondrial stress conditions (Chong et al. 2012). Thus, we evaluated the possible role of these two proteins in the *α*‐CGRP mediated cardio‐protection in this heart failure model. Following four weeks of TAC surgery, an increase in the left ventricular nuclear expression of sirt1 and phosphorylation of AMPK was evident while *α*‐CGRP administration attenuated these augmented levels to those seen in the sham mice. An increased expression of sirt1 in TAC‐hearts has been demonstrated previously (Alcendor et al. 2007; Oka et al. 2011), and overexpression of SIRT1 resulted in increased oxidative stress, apoptosis and fibrosis, and cardiac hypertrophy. In contrast, mice with a mild increase in the expression of sirt1 displayed resistance to oxidative stress and apoptosis in the heart (Alcendor et al. 2007). This implies that mild activation of sirt1 may protect against oxidative stress while greater activation, to levels seen in this present study, may promote oxidative stress. Furthermore, Oka et al. (2011) showed that after four weeks following the TAC procedure, there was greater cardiac expression of sirt1. In these TAC hearts, increased expression of sirt1, in combination with PPAR*α*, suppressed the ERR transcriptional pathway regulation of mitochondrial genes that, in turn, promote mitochondrial dysfunction and the further progression to cardiac hypertrophy and failure. Our results are in agreement with these reports where we observed a marked increase in LV sirt1 levels, oxidative stress, apoptosis and cardiac dysfunction four weeks following TAC pressure‐overload. This suggests that, at this time point, sirt1 may mediate many of the deleterious effects of TAC pressure‐overload by inducing oxidative stress. sirt1 activity is enhanced by AMPK, similarly the activation (phosphorylation) of AMPK occurs either by sirt1 through LKB1, or by high levels of AMP/ATP in energy stress, starvation conditions (Wang et al. 2012). Thus, the TAC LVs activation of AMPK may be due to the pressure‐overload induced energy stress, or by the sirt1/LKB1 pathway. How *α*‐CGRP affects the level of ATP and NAD^+^ in TAC cardiac cells to regulate sirt1 and AMPK expression and activation need further examination. There is no literature presently available to the best of our knowledge showing a direct interaction of sirt1 and *α*‐CGRP. However, sirt1‐transgenic and/or ‐knockout mice would be a good model to conduct these studies.

It has been reported that *α*‐CGRP KO mice have increased activity of the circulating angiotensin‐renin system, and suggested that increased activity of the circulating angiotensin‐renin system might contribute in the further increase in cardiac hypertrophy in TAC *α*‐CGRP KO mice (Li et al. 2013; Li et al. 2004). Angiotensin II is known to stimulate cardiomyocyte hypertrophy (Mazzolai et al. 2000). Therefore the cardio‐protective effect of exogenously delivered *α*‐CGRP, as seen in present report, could be mediated, in part, through angiotensin action and needs further investigation. We are presently investigating this possibility.

As *α*‐CGRP is a potent vasodilator it is possible that the beneficial effects of *α*‐CGRP in TAC mice are due to reduced arterial pressure above the aortic constriction. Our earlier studies in the deoxycorticosterone (DOC)‐salt hypertension mouse model demonstrated that the *α*‐CGRP has a blood pressure (BP)‐independent protective effect on the kidney damage in this model. In this study, blood pressure equalization by hydralazine treatment in DOC‐salt *α*‐CGRP KO mice to levels of the DOC‐salt WT mice did not markedly improve the renal dysfunction seen in the DOC‐salt *α*‐CGRP KO mice which suggested the renal protection was in part or totally BP‐independent (Li et al. 2004). Our another previous dose response study in conscious rats showed that after single intravenous administration of 22, 65, 220, and 2200 pmol of *α*‐CGRP, a significant reduction in mean blood pressure was observed at a dose of 65 pmol, but not at 22 pmol *α*‐CGRP (DiPette et al. 1989). Moreover another study demonstrated that *α*‐CGRP infusion for 7 days through osmotic minipumps at a rate of 1 µg/h (that corresponds to release 0.073 pmole *α*‐CGRP per second) in pregnant rats as well as in L‐NAME induced hypertensive pregnant rats did not reduced blood pressure during postpartum period in both groups of mice (Yallampalli et al. 1996). In addition, a human study conducted with CHF patients showed that patients given a *α‐*CGRP dose of 12.5 µg/h (that corresponds to 3.28 nmole/h or 0.9 pmol/sec), given by intravenous infusion for 24 h, improved myocardial contractility without any consistent change in arterial pressure (Gennari et al. 1990). We calculated that *α*‐CGRP‐filled osmotic minipump used in our present study released ~0.3 pmole of *α‐*CGRP per second systemically. In addition, the ELISA assay performed after 28 days of delivery of *α*‐CGRP through the osmotic pump in TAC mice showed that the LV *α‐*CGRP level in the TAC‐*α*‐CGRP mice was under the physiological range when compared with that of the *α*‐CGRP level in the sham mice (Fig. 1A). Collectively, these reports support our believe that the *α‐*CGRP dose which was administered in the present study would be unlikely to significantly reduce the BP in the vessel distribution proximal to the TAC. Thus, the cardio‐protective action of *α*‐CGRP observed in this study is more likely to be result of a direct cellular effect. However, a partial vasodilatory, BP‐dependent mechanism cannot be excluded.

**Figure 6:**
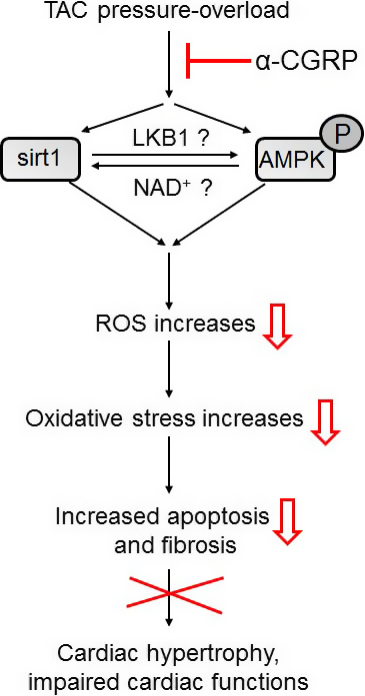
A putative model representing the cardio‐protective effect of *α*‐CGRP in TAC pressure‐overload induced heart failure.

Combining our present results and to those of others has led to the proposal of a putative model through which *α*‐CGRP may protect the heart against pressure‐induced heart failure. TAC‐induced pressure overload and stress, either directly or through AMPK activation, increases the nuclear expression of sirt1 that in combination with PPAR*α* suppresses the ERR transcriptional pathway and impairs mitochondrial biogenesis (as reported by Oka et al. (2011). Dysfunctional mitochondria under stress conditions generate excess reactive oxygen species (ROS), as a result of lipid peroxidation and oxidative DNA damage, which leads to apoptosis and fibrosis. This pathophysiologic response ultimately results in cardiac hypertrophy and subsequent dilation, dysfunction, and failure. In this model the administration of *α*‐CGRP inhibits the expression of sirt1 and phosphorylation of AMPK which, in turn, inhibits ROS generation, apoptosis, and fibrosis resulting in cardio‐protection (as shown by open red arrows in Fig. 6).

Currently, this is the first study demonstrating the beneficial effect of native form of *α*‐CGRP in TAC pressure‐overload induced heart failure. Moreover these results confirm and extend the findings that the administration of an analogue of *α*‐CGRP is cardio‐protective in abdominal aortic constriction‐induced heart failure (Aubdool et al. 2017). Furthermore, our results suggest that the mechanism(s) of the cardio‐protection of *α*‐CGRP in TAC‐induced heart failure is mediated by attenuation of apoptosis and oxidative stress. This attenuation may be mediated through sirt1 and AMPK cellular pathways.

Another study carried out in N^G^‐nitro‐L‐arginine methyl ester (L‐NAME) induced pre‐eclampsia rats demonstrated that 7 days infusion of *α*‐CGRP (1 *µ*g/h) by osmotic minipump significantly improved growth and survival of pups. *α*‐CGRP administration prevented the gestational L‐NAME induced hypertension in pregnant rats, but not during postpartum period, further confirming that osmotic minipumps used to deliver peptide released biologically active *α*‐CGRP in vivo (Yallampalli et al. 1996). However, non‐applicability of osmotic pumps in humans and short half‐life of *α*‐CGRP (~5.5 min in the human plasma (MaassenVanDenBrink et al. 2016) limit this approach to use *α*‐CGRP as a drug in humans. To overcome this problem, novel drug delivery systems for *α*‐CGRP are needed to maintain a constant level of *α*‐CGRP in human plasma for a long‐term treatment regime. Taken together, our studies support *α*‐CGRP as a promising therapeutic agent to treat and possibly prevent cardiovascular diseases, particularly heart failure, ischemic‐reperfusion injury, and hypertension.

## Conflict of Interest

No conflicts of interest, financial or otherwise, are declared by the authors.

## References

[phy214269-bib-0001] Alcendor, R. R. , S. Gao , P. Zhai , D. Zablocki , E. Holle , X. Yu , et al. 2007 Sirt1 regulates aging and resistance to oxidative stress in the heart. Circ. Res. 100:1512–1521. 17446436 10.1161/01.RES.0000267723.65696.4a

[phy214269-bib-0002] Aubdool, A. A. , P. Thakore , F. Argunhan , S. J. Smillie , M. Schnelle , S. Srivastava , et al. 2017 A novel alpha‐Calcitonin gene‐related peptide analogue protects against end‐organ damage in experimental hypertension, cardiac hypertrophy, and heart failure. Circulation 136:367–383. 28446517 10.1161/CIRCULATIONAHA.117.028388 PMC5519346

[phy214269-bib-0003] Bivalacqua, T. J. , A. L. Hyman , P. J. Kadowitz , N. Paolocci , D. A. Kass and H. C. Champion . 2002 Role of calcitonin gene‐related peptide (CGRP) in chronic hypoxia‐induced pulmonary hypertension in the mouse. Influence of gene transfer in vivo. Regul. Pept. 108:129–133. 12220736 10.1016/s0167-0115(02)00100-3

[phy214269-bib-0004] Brain, S. D. , T. J. Williams , J. R. Tippins , H. R. Morris , and I. MacIntyre . 1985 Calcitonin gene‐related peptide is a potent vasodilator. Nature 313:54–56. 3917554 10.1038/313054a0

[phy214269-bib-0005] Breimer, L. H. , I. MacIntyre , and M. Zaidi .1988 Peptides from the calcitonin genes: molecular genetics, structure and function. Biochem. J. 255:377–390. 3060108 10.1042/bj2550377 PMC1135239

[phy214269-bib-0006] Burgoyne, J. R. , H. Mongue‐Din , P. Eaton , and A. M. Shah . 2012 Redox signaling in cardiac physiology and pathology. Circ. Res. 111:1091–1106. 23023511 10.1161/CIRCRESAHA.111.255216

[phy214269-bib-0007] Chai, W. , S. Mehrotra , A. H. Jan Danser , and R. G. Schoemaker . 2006 The role of calcitonin gene‐related peptide (CGRP) in ischemic preconditioning in isolated rat hearts. Eur. J. Pharmacol. 531:246–253. 16438955 10.1016/j.ejphar.2005.12.039

[phy214269-bib-0008] Chong, Z. Z. , S. Wang , Y. C. Shang , and K. Maiese . 2012 Targeting cardiovascular disease with novel SIRT1 pathways. Future Cardiol. 8:89–100. 22185448 10.2217/fca.11.76 PMC3254055

[phy214269-bib-0009] DiPette, D. J. , K. Schwarzenberger , N. Kerr , and O. B. Holland . 1989 Dose‐dependent systemic and regional hemodynamic effects of calcitonin gene‐related peptide. Am. J. Med. Sci. 297:65–70. 2784032 10.1097/00000441-198902000-00001

[phy214269-bib-0010] Dubois‐Rande, J. L. , P. Merlet , C. Benvenuti , S. Sediame , I. Macquin‐Mavier , E. Chabrier , et al. 1992 Effects of calcitonin gene‐related peptide on cardiac contractility, coronary hemodynamics and myocardial energetics in idiopathic dilated cardiomyopathy. Am. J. Cardiol. 70:906–912. 1388329 10.1016/0002-9149(92)90736-i

[phy214269-bib-0011] Franco‐Cereceda, A. , A. Saria , and J. M. Lundberg . 1989 Differential release of calcitonin gene‐related peptide and neuropeptide Y from the isolated heart by capsaicin, ischaemia, nicotine, bradykinin and ouabain. Acta Physiol. Scand. 135:173–187. 2784250 10.1111/j.1748-1716.1989.tb08565.x

[phy214269-bib-0012] Gangula, P. R. , S. C. Supowit , S. J. Wimalawansa , H. Zhao , D. M. Hallman , D. J. DiPette , et al. 1997 Calcitonin gene‐related peptide is a depressor in NG‐nitro‐L‐arginine methyl ester‐induced hypertension during pregnancy. Hypertension 29:248–253. 9039110 10.1161/01.hyp.29.1.248

[phy214269-bib-0013] Gennari, C. , R. Nami , D. Agnusdei , and J. A. Fischer . 1990 Improved cardiac performance with human calcitonin gene related peptide in patients with congestive heart failure. Cardiovasc. Res. 24:239–241. 2346957 10.1093/cvr/24.3.239

[phy214269-bib-0014] Huang, R. , A. Karve , I. Shah , M. C. Bowers , D. J. DiPette , S. C. Supowit , et al. 2008 Deletion of the mouse alpha‐calcitonin gene‐related peptide gene increases the vulnerability of the heart to ischemia‐reperfusion injury. Am. J. Physiol. Heart Circ. Physiol. 294:H1291–H1297. 18192222 10.1152/ajpheart.00749.2007

[phy214269-bib-0015] Kannan, K. , and S. K. Jain . 2000 Oxidative stress and apoptosis. Pathophysiology 7:153–163. 10996508 10.1016/s0928-4680(00)00053-5

[phy214269-bib-0016] Katki, K. A. , S. C. Supowit , and D. J. DiPette . 2001 Role of calcitonin gene‐related peptide and substance P in Dahl‐salt hypertension. Hypertension 38:679–682. 11566955 10.1161/hy09t1.095761

[phy214269-bib-0017] Kumar, A. , C. K. Singh , H. A. Lavoie , D. J. Dipette , and U. S. Singh . 2011 Resveratrol restores Nrf2 level and prevents ethanol‐induced toxic effects in the cerebellum of a rodent model of fetal alcohol spectrum disorders. Mol. Pharmacol. 80:446–457. 21697273 10.1124/mol.111.071126 PMC3164333

[phy214269-bib-0018] Kumar, A. , N. Al‐Sammarraie , D. J. DiPette , and U. S. Singh . 2014 Metformin impairs Rho GTPase signaling to induce apoptosis in neuroblastoma cells and inhibits growth of tumors in the xenograft mouse model of neuroblastoma. Oncotarget 5:11709–11722. 25365944 10.18632/oncotarget.2606 PMC4294363

[phy214269-bib-0019] Li, J. , H. Zhao , S. C. Supowit , D. J. DiPette , and D. H. Wang . 2004 Activation of the renin‐angiotensin system in alpha‐calcitonin gene‐related peptide/calcitonin gene knockout mice. J. Hypertens. 22:1345–1349. 15201551 10.1097/01.hjh.0000125409.50839.f1

[phy214269-bib-0020] Li, J. , T. Ichikawa , L. Villacorta , J. S. Janicki , G. L. Brower , M. Yamamoto , et al. 2009 Nrf2 protects against maladaptive cardiac responses to hemodynamic stress. Arterioscler. Thromb. Vasc. Biol. 29:1843–1850. 19592468 10.1161/ATVBAHA.109.189480 PMC12952473

[phy214269-bib-0021] Li, J. , S. P. Levick , D. J. DiPette , J. S. Janicki , and S. C. Supowit . 2013 Alpha‐calcitonin gene‐related peptide is protective against pressure overload‐induced heart failure. Regul. Pept. 185:20–28. 23816470 10.1016/j.regpep.2013.06.008

[phy214269-bib-0022] Li, J. , K. A. Carnevale , D. J. Dipette , and S. C. Supowit .2013 Renal protective effects of alpha‐calcitonin gene‐related peptide in deoxycorticosterone‐salt hypertension. Am. J. Physiol. Renal Physiol. 304:F1000–F1008. 23389451 10.1152/ajprenal.00434.2012

[phy214269-bib-0023] MaassenVanDenBrink, A. , J. Meijer , C. M. Villalon , and M. D. Ferrari . 2016 Wiping Out CGRP: Potential Cardiovascular Risks. Trends Pharmacol. Sci. 37:779–788. 27338837 10.1016/j.tips.2016.06.002

[phy214269-bib-0024] Mazzolai, L. , T. Pedrazzini , F. Nicoud , G. Gabbiani , H. R. Brunner , and J. Nussberger . 2000 Increased cardiac angiotensin II levels induce right and left ventricular hypertrophy in normotensive mice. Hypertension 35:985–991. 10775573 10.1161/01.hyp.35.4.985

[phy214269-bib-0025] Nakamura, M. , and J. Sadoshima . 2018 Mechanisms of physiological and pathological cardiac hypertrophy. Nat. Rev. Cardiol. 15:387–407. 29674714 10.1038/s41569-018-0007-y

[phy214269-bib-0026] Nakayama, H. , X. Chen , C. P. Baines , R. Klevitsky , X. Zhang , H. Zhang , et al. 2007 Ca2+‐ and mitochondrial‐dependent cardiomyocyte necrosis as a primary mediator of heart failure. J. Clin. Investig. 117:2431–2444. 17694179 10.1172/JCI31060 PMC1937500

[phy214269-bib-0027] Niture, S. K. , R. Khatri , and A. K. Jaiswal . 2014 Regulation of Nrf2‐an update. Free Radic. Biol. Med. 66:36–44. 23434765 10.1016/j.freeradbiomed.2013.02.008 PMC3773280

[phy214269-bib-0028] Nojiri, H. , T. Shimizu , M. Funakoshi , O. Yamaguchi , H. Zhou , S. Kawakami , et al. 2006 Oxidative stress causes heart failure with impaired mitochondrial respiration. J. Biol. Chem. 281:33789–33801. 16959785 10.1074/jbc.M602118200

[phy214269-bib-0029] Oka, S. , R. Alcendor , P. Zhai , J. Y. Park , D. Shao , J. Cho , et al. 2011 PPARalpha‐Sirt1 complex mediates cardiac hypertrophy and failure through suppression of the ERR transcriptional pathway. Cell Metab. 14:598–611. 22055503 10.1016/j.cmet.2011.10.001 PMC3217210

[phy214269-bib-0030] van der Pol, A. , W. H. van Gilst , A. A. Voors , and P. van der Meer . 2019 Treating oxidative stress in heart failure: past, present and future. Eur. J. Heart Fail. 21:425–435. 30338885 10.1002/ejhf.1320 PMC6607515

[phy214269-bib-0031] Rosenfeld, M. G. , J. J. Mermod , S. G. Amara , L. W. Swanson , P. E. Sawchenko , J. Rivier , et al. 1983 Production of a novel neuropeptide encoded by the calcitonin gene via tissue‐specific RNA processing. Nature 304:129–135. 6346105 10.1038/304129a0

[phy214269-bib-0032] Russell, L. K. , B. N. Finck , and D. P. Kelly . 2005 Mouse models of mitochondrial dysfunction and heart failure. J. Mol. Cell. Cardiol. 38:81–91. 15623424 10.1016/j.yjmcc.2004.10.010

[phy214269-bib-0033] Russell, F. A. , R. King , S. J. Smillie , X. Kodji , and S. D. Brain . 2014 Calcitonin gene‐related peptide: physiology and pathophysiology. Physiol. Rev. 94:1099–1142. 25287861 10.1152/physrev.00034.2013 PMC4187032

[phy214269-bib-0034] Shekhar, Y. C. , I. S. Anand , R. Sarma , R. Ferrari , P. L. Wahi , and P. A. Poole‐Wilson . 1991 Effects of prolonged infusion of human alpha calcitonin gene‐related peptide on hemodynamics, renal blood flow and hormone levels in congestive heart failure. Am. J. Cardiol. 67:732–736. 2006623 10.1016/0002-9149(91)90531-o

[phy214269-bib-0035] Supowit, S. C. , C. V. Ramana , K. N. Westlund , and D. J. DiPette . 1993 Calcitonin gene‐related peptide gene expression in the spontaneously hypertensive rat. Hypertension 21:1010–1014. 8505084 10.1161/01.hyp.21.6.1010

[phy214269-bib-0036] Supowit, S. C. , H. Zhao , D. H. Wang , and D. J. DiPette . 1995 Regulation of neuronal calcitonin gene‐related peptide expression. Role of increased blood pressure. Hypertension 26:1177–1180. 7498991 10.1161/01.hyp.26.6.1177

[phy214269-bib-0037] Supowit, S. C. , H. Zhao , D. M. Hallman , and D. J. DiPette . 1997 Calcitonin gene‐related peptide is a depressor of deoxycorticosterone‐salt hypertension in the rat. Hypertension 29:945–950. 9095081 10.1161/01.hyp.29.4.945

[phy214269-bib-0038] Supowit, S. C. , H. Zhao , D. M. Hallman , and D. J. DiPette . 1998 Calcitonin gene‐related peptide is a depressor in subtotal nephrectomy hypertension. Hypertension 31:391–396. 9453334 10.1161/01.hyp.31.1.391

[phy214269-bib-0039] Supowit, S. C. , A. Rao , M. C. Bowers , H. Zhao , G. Fink , B. Steficek , et al. 2005 Calcitonin gene‐related peptide protects against hypertension‐induced heart and kidney damage. Hypertension 45:109–114. 15583078 10.1161/01.HYP.0000151130.34874.fa

[phy214269-bib-0040] Wang, S. , P. Song , and M. H. Zou . 2012 AMP‐activated protein kinase, stress responses and cardiovascular diseases. Clin. Sci. 122:555–573. 22390198 10.1042/CS20110625 PMC3367961

[phy214269-bib-0041] Wharton, J. , S. Gulbenkian , P. K. Mulderry , M. A. Ghatei , G. P. McGregor , S. R. Bloom , et al. 1986 Capsaicin induces a depletion of calcitonin gene‐related peptide (CGRP)‐immunoreactive nerves in the cardiovascular system of the guinea pig and rat. J. Auton. Nerv. Syst. 16:289–309. 2427561 10.1016/0165-1838(86)90035-4

[phy214269-bib-0042] Yallampalli, C. , Y. L. Dong , and S. J. Wimalawansa . 1996 Calcitonin gene‐related peptide reverses the hypertension and significantly decreases the fetal mortality in pre‐eclampsia rats induced by NG‐nitro‐Lmethyl ester. Hum. Reprod. 11:895–899. 8671348

[phy214269-bib-0043] Zhang, R. L. , Z. Guo , L. L. Wang , and J. Wu . 2012 Degeneration of capsaicin sensitive sensory nerves enhances myocardial injury in acute myocardial infarction in rats. Int. J. Cardiol. 160:41–47. 21470700 10.1016/j.ijcard.2011.03.025

